# Can greenspace modify the combined effects of multiple air pollutants on pulmonary tuberculosis treatment outcomes? An empirical study conducted in Zhejiang Province, China

**DOI:** 10.1265/ehpm.24-00381

**Published:** 2025-05-02

**Authors:** Bo Xie, Maolin Wu, Zhe Pang, Bin Chen

**Affiliations:** 1 School of Urban Design, Wuhan University, Wuhan, China; 2 Zhejiang Provincial Center for Disease Control and Prevention, Hangzhou, China

**Keywords:** Pulmonary tuberculosis treatment, Combined air pollutants, Greenspace, Combined effect, Effect modification

## Abstract

**Background:**

Evidence on the combined effects of air pollutants and greenspace exposure on pulmonary tuberculosis (PTB) treatment is limited, particularly in developing countries with high levels of air pollution.

**Objective:**

We aimed to examine the individual and combined effects of long-term exposure to air pollutants on PTB treatment outcomes while also investigating the potential modifying effect of greenspace.

**Methods:**

This population-based study included 82,784 PTB cases notified in Zhejiang Province, China, from 2015 to 2019. The 24-month average concentrations of particulate matter with an aerodynamic diameter ≤2.5 µm (PM_2.5_), ozone (O_3_), nitrogen dioxide (NO_2_), and sulfur dioxide (SO_2_) before PTB diagnosis were estimated using a dataset derived from satellite-based machine learning models and monitoring stations. Greenspace exposure was assessed using the annual China Land Cover Dataset. We conducted analyses using time-varying Cox proportional hazards models and cumulative risk indices.

**Results:**

In individual effect models, each 10 µg/m^3^ increase in PM_2.5_, NO_2_, O_3_, and SO_2_ concentrations was associated with hazard ratios for PTB treatment success of 0.95 (95% confidence interval (CI): 0.93–0.97), 0.92 (95% CI: 0.91–0.94), 0.98 (95% CI: 0.97–0.99), and 1.52 (95% CI: 1.49–1.56), respectively. In combined effect models, long-term exposure to the combination of air pollutants was negatively associated with PTB treatment success, with a joint hazard ratio (JHR) of 0.79 (95% CI: 0.63–0.96). Among the pollutants examined, O_3_ contributed the most to the increased risks, followed by PM_2.5_ and NO_2_. Additionally, areas with moderate levels of greenspace showed a reduced risk (JHR = 0.81, 95% CI: 0.62–0.98) compared with the estimate from the third quantile model (JHR = 0.68, 95% CI: 0.52–0.83).

**Conclusions:**

Combined air pollutants significantly impede successful PTB treatment outcomes, with O_3_ and PM_2.5_ accounting for nearly 75% of this detrimental effect. Moderate levels of greenspace can mitigate the adverse effects associated with combined air pollutants, leading to improved treatment success for patients with PTB.

**Supplementary information:**

The online version contains supplementary material available at https://doi.org/10.1265/ehpm.24-00381.

## 1. Introduction

Pulmonary tuberculosis (PTB), caused by *Mycobacterium tuberculosis* (*M.tb*), remains a significant global public health challenge, particularly in developing countries [1]. In 2022, approximately USD 5.8 billion was allocated to PTB prevention, diagnosis, and treatment programs across 128 low- and middle-income countries [2]. Additionally, increased local financial support has improved access to free tuberculosis (TB) treatments and effective medications [3]. Despite these efforts, the global treatment success rate for new and relapse cases of PTB remains at 85% [4]. To further reduce transmission and mortality rates, it is essential to identify modifiable environmental risk factors and integrate them into comprehensive intervention strategies. Combining these strategies with pharmacological treatments and financial subsidies could enhance the cost-effectiveness of public health initiatives.

Recent evidence highlights ambient air pollution as a significant risk factor influencing PTB treatment outcomes. Laboratory-based studies have shown that air pollutants can increase susceptibility to *M.tb* infection through mechanisms such as oxidative damage and inflammatory responses [5]. Epidemiological studies further reveal that long-term exposure to high levels of particulate matter with an aerodynamic diameter ≤2.5 µm (PM_2.5_), nitrogen dioxide (NO_2_), and ozone (O_3_) is associated with increased PTB mortality [6, 7]. In contrast, some studies suggest that short-term exposure to sulfur dioxide (SO_2_) may have a protective effect against PTB [8, 9]. The implementation of air pollution control measures has emerged as a prioritized and cost-effective strategy for reducing PTB risks, which builds on the growing body of evidence. Additionally, the critical role of greenspaces in alleviating health-related burdens is gaining recognition, as they serve as natural filters for air pollution [10, 11]. Several studies have reported an inverse association between higher levels of neighborhood greenspace and the incidence and mortality of PTB related to specific pollutants, such as PM_2.5_ [12–15]. However, current research on the complex interplay between air pollution, greenspace exposure, and PTB risks faces three key limitations.

First, most existing research has focused on individual PTB treatment outcomes, particularly mortality [16, 17]. However, there is limited epidemiological evidence examining the associations between air pollutants and multiple PTB treatment outcomes. To date, only one published study has explored the relationship between outdoor air pollution and TB treatment success, which specifically addresses sputum culture conversion. However, this study was limited to particulate matter with an aerodynamic diameter ≤10 µm (PM_10_) [18]. Considering that patients with PTB are often exposed to complex mixtures of air pollutants during long-term treatment and healthcare services, it is essential to investigate the associations between a broader range of pollutants and diverse PTB treatment outcomes.

Second, most prior studies have focused on assessing the individual effects of single air pollutants, which often treat other pollutants as covariates to ensure robustness in their evaluations [19, 20]. However, there is a substantial knowledge gap regarding the combined effects of air pollutant mixtures on PTB treatment outcomes. Within these mixtures, pollutants may interact synergistically or antagonistically, which amplifies or mitigates their impacts. For example, particulate matter can physically adsorb gases, which potentially increases the inhalation dose of pollutants and facilitates the reactivation of *M.tb*. Furthermore, co-exposure scenarios can trigger chemical reactions among various air pollutants and generate secondary compounds that exacerbate immune system impairment and elevate the risk of *M.tb* activation [21].

Third, existing research has predominantly examined the independent associations between greenspace and specific air pollutants and overlooked greenspaces’ potential to mitigate the combined adverse effects of exposure to multiple pollutants. The extent to which greenspace alleviates these cumulative effects remains unclear. Notably, greenspace may influence different air pollutants in varying ways, potentially leading to health benefits that counteract one another. For example, while vegetation has been shown to effectively reduce concentrations of PM_2.5_ and NO_2_ [22, 23], it can also contribute to elevated O_3_ levels due to the emission of biogenic volatile organic compounds [24, 25]. This increase in O_3_ concentrations associated with vegetation could offset some of the health benefits gained from the reduction in PM_2.5_ and NO_2_ levels. Furthermore, greenspace may modify interactions between air pollutants by creating a localized microclimate [26]. Vegetation regulates air temperature through processes such as transpiration and the interception of solar radiation [27, 28], which can influence the chemical reaction rates of air pollutants. Consequently, the modifying effects of greenspace on multiple air pollutants may alter the immune responses of patients to *M.tb* reactivation, resulting in outcomes that differ from those observed with individual pollutants.

To address these gaps, we aimed to (1) investigate the individual and combined effects of long-term exposure to ambient air pollutants, including PM_2.5_, SO_2_, NO_2_, and O_3_, on PTB treatment outcomes in Zhejiang Province, China; and (2) to explore the potential modifying effects of residential greenspace on the associations between air pollutant mixtures and PTB treatment outcomes.

## 2. Materials and methods

### 2.1 Study population

We collected data on PTB cases from January 1, 2015, to December 31, 2019, in Zhejiang Province, China. The dataset included information on patients with PTB such as sex, age, residential address, diagnosis details, and occupation. All data were extracted from the Web-based Tuberculosis Information Management System (TBIMS) in China [29, 30]. This system records all notified TB cases at county, city, and provincial levels across various hospitals, with subsequent verification by the Zhejiang Provincial Center for Disease Control and Prevention. Additionally, clinical details, including the date of diagnosis, treatment regimen, etiological test results, and treatment outcomes, were collected. The “event date” for analysis was defined as the date of diagnosis. Geocoding was performed using the registered home addresses of patients to determine individual exposure levels. To ensure accuracy, patients with incorrect or invalid addresses were excluded from the analysis.

**Fig. 1:**
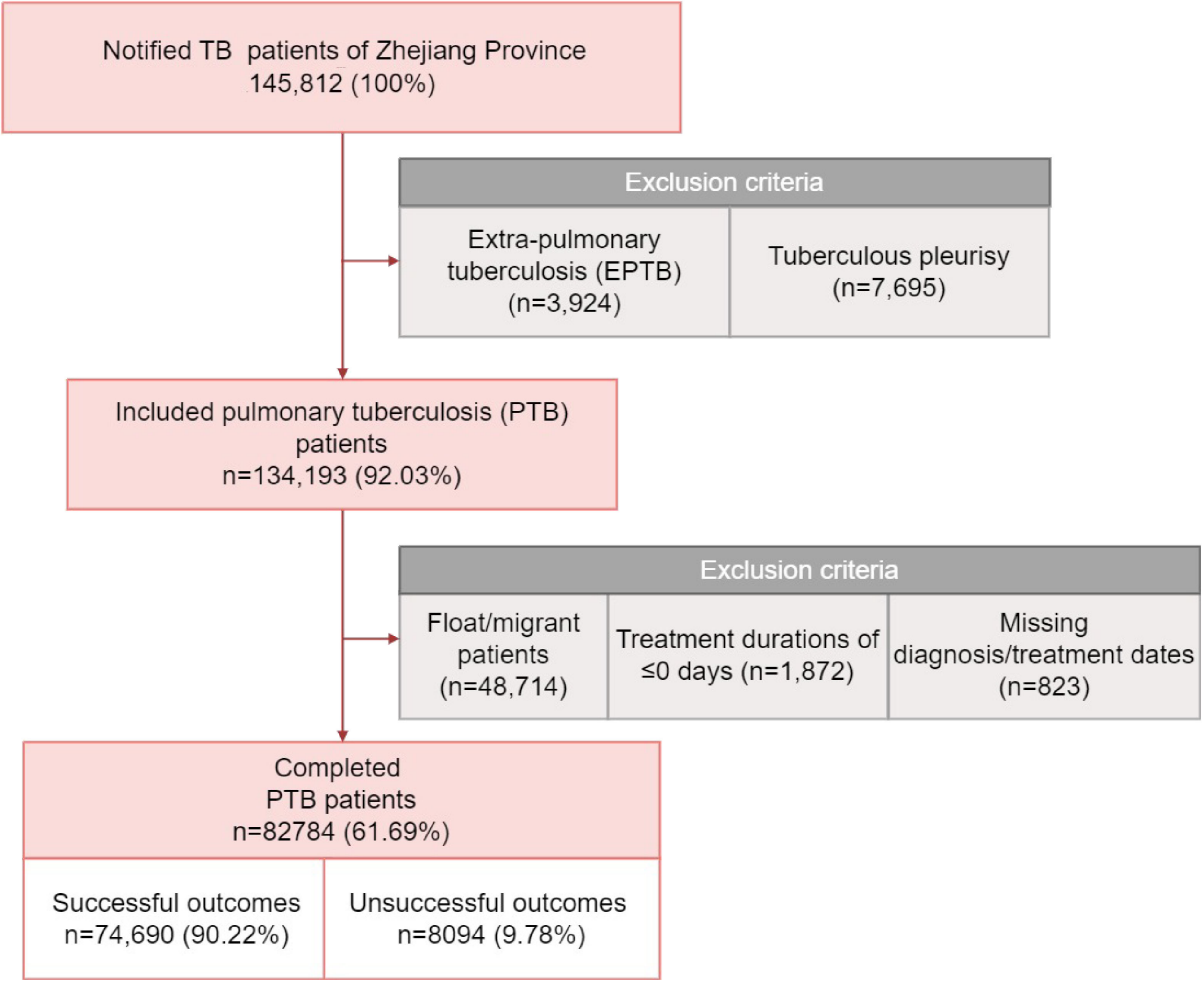
Flow diagram of patient exclusions and retention.

First, a total of 145,812 individuals were diagnosed with TB. We excluded patients with extra pulmonary TB (n = 3,924), tuberculous pleurisy (n = 7,695), missing diagnosis or treatment dates (n = 823), and treatment durations of ≤0 days (n = 1,872). Additionally, floating and migrating populations were excluded (n = 48,714) to minimize residential self-selection biases and ensure a more accurate assessment of the impact of long-term environmental exposure on PTB treatment outcomes [10]. We compared the baseline characteristics between the group including migrants (n = 131,498) and the group excluding migrants (n = 82,784) using the Chi-square test to ensure representativeness (Table S1). Ultimately, a total of 82,784 PTB cases in Zhejiang Province were included in our analysis (Fig. 1).

### 2.2 Outcome definition

***Outcome event.*** The TBIMS includes notified PTB cases classified according to the National Diagnostic Criteria for Pulmonary Tuberculosis (WS288-2008, WS288-2017) [31, 32]. Treatment outcomes for all PTB cases were categorized as successful (including “cured” and “treatment completed”) or unsuccessful (including “died,” “treatment failed,” and “loss to follow-up”). Specifically, a successful outcome was defined as completing the prescribed therapeutic regimen and achieving negative sputum smear or culture results at the end of treatment. Unsuccessful outcomes included death during treatment, failure of the treatment protocol to produce the desired effects, or non-compliance with required follow-ups. Detailed definitions of these treatment outcomes are provided in Table S2.

***Survival time.*** Survival time was defined as the duration from the date of PTB diagnosis to the completion of treatment. For patients who did not complete treatment (e.g., died, treatment failed, or loss to follow-up), survival time was calculated as the duration from the date of diagnosis to the last recorded registration.

### 2.3 Exposure assessment

***Air pollution.*** The monthly average concentrations of PM_2.5_ (24-hour), SO_2_ (24-hour), NO_2_ (24-hour), and ground-level O_3_ (8-hour) were obtained from the high-resolution “*China High Air Pollutants*” dataset [33–35]. This dataset provided PM_2.5_ and O_3_ data at a spatial resolution of 1 km, while for NO_2_ and SO_2_, the resolution was initially 10 km from 2015 to 2018 but was upgraded to 1 km in 2019. The dataset’s cross-validation R^2^ ranges from 0.80 to 0.93, with a root-mean-square error between 4.89 and 24.28 µg/m^3^, which indicates high data quality [35, 36]. Given the long-term latency period following *M.tb* infection, a 24-month exposure window was selected to comprehensively capture the cumulative immunomodulatory effects of environmental pollutants on host defenses against bacterial reactivation [37, 38]. Furthermore, considering that standard anti-tuberculosis treatment typically lasts 6–8 months (and can extend to 9–24 months for drug-resistant cases [39]), sustained pollutant exposure during this period may persistently influence treatment efficacy through pharmacological interference or adherence modulation. Therefore, we defined the exposure window as covering both the 24 months preceding the diagnosis date and the entire treatment period.

***Greenspace.*** Greenspace, defined as the cumulative proportion of cropland, forest, grassland, and shrub within 1,250 m buffer zones, was derived from the China Land Cover Dataset at a resolution of 30 m (https://doi.org/10.5281/zenodo.8176941). The selection of a 1,250 m buffer zone captures greenspace exposure during activities such as walking or exercising near residential areas and within the surrounding environment [40]. The time window for greenspace exposure aligns with that used for evaluating air pollutants.

### 2.4 Covariates

Covariates were grouped into four primary domains: demographic variables, patient characteristics and treatment modalities, socioeconomic status, and environmental factors. Demographic variables included age at diagnosis and sex. Patient characteristics and treatment modalities encompassed treatment history, etiological test results, and drug resistance status. Patients were classified into initial and retreatment groups based on their treatment history. The initial treatment group included individuals who had not yet started treatment, those in the early stages of chemotherapy, or those who had received irregular treatment lasting less than a month. In contrast, the retreatment group consisted of patients with a history of irregular treatment or those who experienced relapse after initial treatment failure. The etiological classification was based on pathogen detection in clinical specimens to categorize patients as smear-negative or smear-positive. Drug resistance for *M.tb* was determined through *in vitro* testing to identify individuals with or without resistance to anti-TB medications. Socioeconomic status was assessed based on occupational classification to differentiate physically labor-intensive jobs with lower skill or capital requirements from knowledge-intensive roles that demand advanced education, specialized knowledge, and analytical or creative abilities. The environmental factors considered in this study included the work environment (indoor vs. outdoor), mean temperature, and population density. Temperature data were obtained from the monthly raster dataset of the National Tibetan Plateau Data Center at a 1 km resolution (https://data.tpdc.ac.cn/home), while population density data were sourced from the WorldPop grid layer (https://hub.worldpop.org/). The methods used to calculate mean temperature and average population density were consistent with those employed in air pollutant exposure assessments.

### 2.5 Statistical analysis

Time-varying Cox proportional hazards models were used to investigate the association between long-term exposure to ambient air pollutants (PM_2.5_, NO_2_, SO_2_, and O_3_) and PTB treatment outcomes. We applied a log-transformed time scale (t + 20) as recommended in previous studies [36]. The attained age at baseline and at the end of follow-up was used as the time scale in the Cox proportional hazards regression model [41], inherently adjusting for age during follow-up duration. The proportional hazards assumption was assessed through the Schoenfeld residual method, with all P values >0.05. Adjusted hazard ratios (HRs) with 95% confidence intervals (95% CIs) were calculated for each 10 µg/m^3^ increase in PM_2.5_, NO_2_, SO_2_, and O_3_ concentrations. To address potential confounding, we employed a four-stage modeling approach with progressively adjusted covariates. Specifically, Model 1 included only air pollutants without any adjustments. Model 2 adjusted for demographic factors such as age and sex. Model 3 further controlled for individual clinical variables, including treatment type, drug susceptibility, and pathogen results. Finally, Model 4 incorporated additional factors such as meteorological factors, socioeconomic status, occupation type, and work environment, representing the fully adjusted comprehensive model.

Furthermore, we used the cumulative risk index (CRI) to assess the combined risk of exposure to multiple air pollutants on PTB treatment outcomes. The joint hazard ratios (JHRs) quantified the risks associated with a 10 µg/m^3^ increase in all four air pollutants [42, 43]. The JHRs were derived by combining the *p* exposures evaluated at *x* as the CRI, which was defined as

$$\begin{array}{rl} \mathrm{CRI} & =\exp\left(\sum_{p=1}^{p}{\hat{\beta }}_{p}x_{p}\right)=\exp({\hat{\beta }}^{\prime }x^{\prime })\\ & =\prod_{p=1}^{p}{\text{Joint hazard ratio}}_{p} \end{array}$$

where ${\hat{\beta }}^{\prime }=({\hat{\beta }}_{1},\ldots {\hat{\beta }}_{p})$ represent the estimates of the log-hazard ratios for the *p* pollutants in a Cox survival model that includes all *p* exposures together, $x^{\prime }=(x_{1},\ldots ,x_{p})$ are the levels at which each exposure-specific HR is evaluated, and the *Joint hazard ratio_p_* denotes the JHR for the *p^th^* exposure in a multi-exposure model. JHRs are estimated assuming additive effects of joint exposures. The 95% CI of the CRI is defined by

$$\mathrm{CI}=\exp({\hat{\beta }}^{\prime }x\pm 1.96\times \sqrt{\hat{\beta }\times \mathrm{Cov}(\hat{\beta })\times {\hat{\beta }}^{\prime }})$$

A three-level categorical variable was created to investigate the potential impact of greenspace exposure on the combined effect of multiple air pollutants on PTB treatment outcomes. This variable classified patients into low, medium, and high greenspace exposure groups based on the proportion of greenspace within 1,250 m radius buffers. The statistical significance of our findings was assessed through the likelihood ratio test at a significance level of 0.05, with corresponding p-values reported.

The robustness of the results was assessed through a series of sensitivity analyses. First, additional adjustments were made for different exposure buffer sizes of air pollutants (500 m and 1,250 m buffer radii). Second, a sensitivity analysis was conducted by excluding drug-resistant patients from the dataset to explore potential bias arising from the specialized treatment management required for this subgroup. Third, we compared the primary outcomes estimated by greenspace exposure across 500 m buffer size. Fourth, migrant populations were retained for the reanalysis of baseline characteristics and model validation. Fifth, green spaces were redefined by excluding agricultural land, thereby focusing on forest and grassland coverage within 1,250 m residential buffers. This approach minimizes potential confounding from agrochemical exposure while preserving ecologically meaningful vegetation metrics. All analyses were performed using R (version 4.3.0).

## 3. Results

### 3.1 Descriptive statistics

Table 1 presents the demographic characteristics of the patients. Among the 82,784 cases, 57,977 (70.03%) were male, with a mean age of 54 years (SD ± 19.32), while 24,807 (29.97%) were female, with a mean age of 50 years (SD ± 20.26). A significant proportion of patients (42.91%) were aged 60 years or older. Additionally, 41,466 cases (50.09%) were diagnosed with smear-positive PTB. The majority of patients (89.83%) received initial treatment for PTB upon diagnosis, and *M.tb* resistance was identified in a small subset (2,170, 2.62%). Most participants were engaged in outdoor occupations (72.07%), and a large proportion performed labor-intensive work (83.00%). Moreover, an impressive treatment success rate of 90.22% was achieved, with an average treatment duration of approximately 246 days. Additionally, the Chi-square test comparing the migrant-included and migrant-excluded groups indicated minimal differences in baseline characteristics, thereby suggesting a low likelihood of selection bias and reinforcing the robustness of our findings (Table S1).

**Table 1:** Characteristics of individuals across the tertiles of greenspace exposure.

**Characteristics**	**Greenspace tertile**			

	**All patients ** **(range 0.00%–100.00%)**	**Tertile 1 ** **(range 2%–32.43%)**	**Tertile 2 ** **(range 32.43%–79.04%)**	**Tertile 3 ** **(range 79.04%–100.00%)**
	**n (%)**	**n (%)**	**n (%)**	**n (%)**
**Sex**				
Male	57,977 (70.03%)	18,307 (66.34%)	19,438 (70.44%)	20,232 (73.32%)
Female	24,807 (29.97%)	9,288 (33.66%)	8,157 (29.56%)	7,362 (26.68%)
**Age**				
<=18	2,780 (3.36%)	728 (2.64%)	666 (2.41%)	490 (1.78%)
>18, <=60	44,483 (53.73%)	17,479 (63.34%)	14,694(53.25%)	13,206 (47.86%)
>60	35,521 (42.91%)	9,388 (34.02%)	12,235(44.34%)	13,898 (50.37%)
**Occupation**				
Labor-Intensive	68,714 (83.00%)	19,157 (69.42%)	23,980(86.90%)	25,577 (92.69%)
Knowledge-Intensive	14,070 (17.00%)	8,438 (30.58%)	3,615(13.10%)	2,017 (7.31%)
**Work Environment**				
Indoor	23,120 (27.93%)	12,167 (44.09%)	6,806(24.66%)	4,147 (15.03%)
Outdoor	59,664 (72.07%)	15,428 (55.91%)	20,789(75.34%)	23,447 (84.97%)
**Treatment History**				
Initial treatment	74,367 (89.83%)	25,048 (90.77%)	24,772 (89.77%)	24,547 (88.96%)
Retreatment	8,417 (10.17%)	2,547 (9.23%)	2,823 (10.23%)	3,047 (11.04%)
**Pathogenic results**				
Smear-negative	41,318 (49.91%)	14,924 (54.08%)	13,555 (49.12%)	12,839 (46.53%)
Smear-positive	41,466 (50.09%)	12,671 (45.92%)	14,040 (50.88%)	14,755 (53.47%)
**Drug-susceptibility**				
Drug resistance	2,170 (2.62%)	753 (2.73%)	731 (2.65%)	686 (2.49%)
Non-drug resistance	80,614 (97.38%)	26,842 (97.27%)	26,864 (97.35%)	26,908 (97.51%)
**Treatment time (mean)**	246.31 (±94.54)	251.64 (±97.30)	245.14 (±94.09)	242.16 (±91.90)

**Fig. 2:**
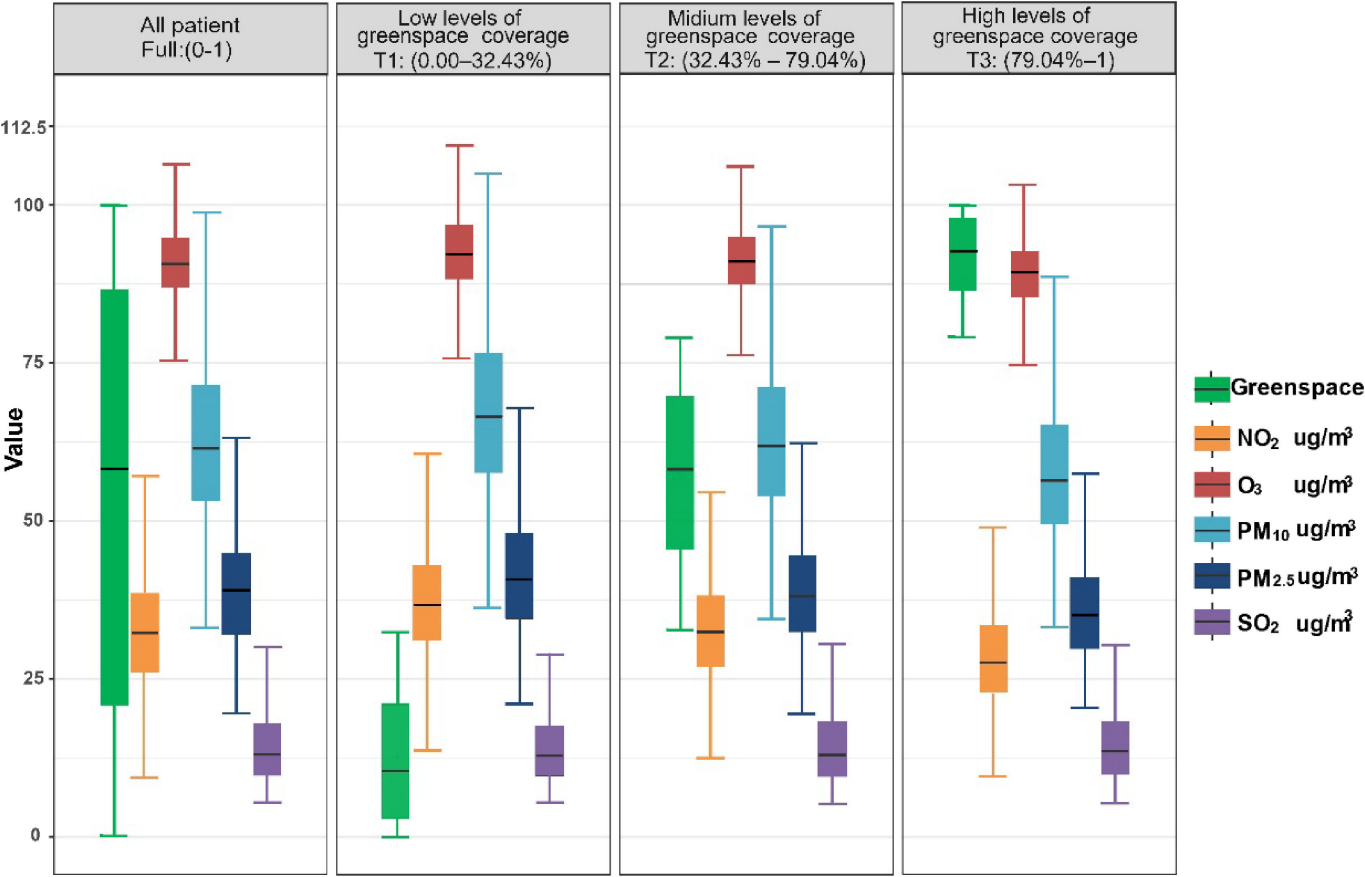
Air pollution exposure statistics by tertiles of greenspace.

The statistical distribution of air pollution across the three tertiles of greenspace is shown in Fig. 2 (T1: 0.00%–32.43%, T2: 32.43%–79.04%, and T3: 79.04%–100%). The average greenspace coverage within a 1,250 m radius was approximately 61.87%. Air pollutant concentrations ranged from 5.35 µg/m^3^ (SO_2_) to 122.03 µg/m^3^ (O_3_), with O_3_ exhibiting the highest average concentration at 91.28 µg/m^3^ and SO_2_ displaying the lowest average concentration at 14.31 µg/m^3^. The proportion of greenspace increased across the tertiles and peaked at 94.02% in the third tertile. Mean concentrations of PM_2.5_, NO_2_, and O_3_ decreased across the tertiles, with NO_2_ showing the most significant reduction of 12.04% (Table S3). Additionally, Spearman correlation analysis revealed a highly significant collinearity between PM_10_ and PM_2.5_ (Sc = 0.99, exceeding the 0.8 threshold; see Fig. S1). After excluding PM_10_ from the analysis, the variance inflation factors for all remaining pollutants were below 10.0, ensuring model stability (Table S4).

### 3.2 Independent effect of air pollution on PTB treatment

**Fig. 3:**
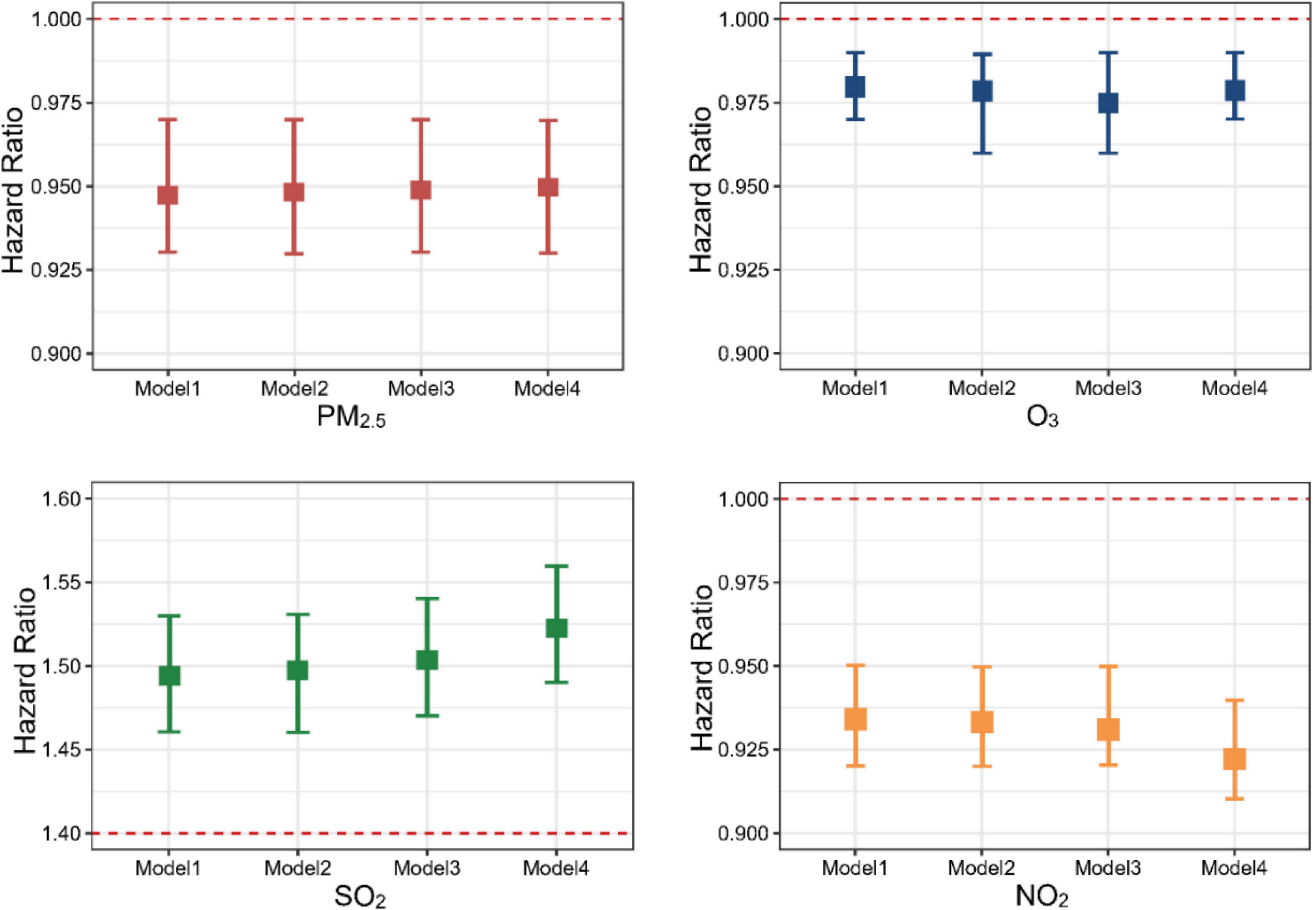
Associations between individual air pollutants and PTB treatment outcomes in multi-exposure models (continuous terms). Notes: Model 1 included only air pollutants without any adjustments. Model 2 adjusted for demographic factors such as age and sex. Model 3 further controlled for individual clinical variables, including treatment type, drug susceptibility, and pathogen results. Finally, Model 4 incorporated additional factors such as meteorological factors, socioeconomic status, occupation type, and work environment, representing the fully adjusted comprehensive model.

The independent effects of air pollutants on PTB treatment outcomes are presented in Fig. 3. The HRs indicate a negative association of PTB treatment success with PM_2.5_ (HR = 0.95, 95% CI: 0.93–0.97), O_3_ (HR = 0.98, 95% CI: 0.97–0.99), and NO_2_ (HR = 0.92, 95% CI: 0.91–0.94) in the continuous terms model. Specifically, a 10 µg/m^3^ increase in PM_2.5_, O_3_, and NO_2_ concentrations was associated with a 5%, 2%, and 8% increased risk of unsuccessful PTB treatment, respectively. Significant adverse associations were also observed in the quintile model, particularly for NO_2_ and O_3_ (Table S5). Conversely, a positive association was found between SO_2_ and PTB treatment success, both in the continuous term model (HR = 1.52, 95% CI: 1.49–1.56, Fig. 3) and the quintile model (Table S5). These associations between air pollution exposure and PTB treatment outcomes remained robust even after adjusting for the buffer size used to estimate pollutant exposures (Table S6). Additionally, the exclusion of drug-resistant patients and the retention of immigrant individuals did not alter these findings, as shown in Table S7 and Table S8.

### 3.3 Combined effect of multiple air pollutants on PTB treatment

The combined effect analysis demonstrated a significant negative impact of combined air pollutants on PTB treatment outcomes (Table 2). Specifically, for each 10 µg/m^3^ increase in the concentration of the four pollutants, the risk of unsuccessful PTB treatment increased by 21% (JHR = 0.79, 95% CI: 0.63–0.96). Among these pollutants (Model 4 in Table 2), O_3_ had the most significant adverse effect (51.52%), followed by PM_2.5_ (22.02%) and NO_2_ (18.38%). We conducted three models with stepwise adjustments for potential confounding factors and consistently observed these associations (Models 1, 2, and 3 in Table 2).

**Table 2:** JHRs and degree of contribution (DOC) of air pollutants.

**Variable**	**Model 1**		**Model 2**		**Model 3**		**Model 4**	

	**JHR**	**DOC**	**JHR**	**DOC**	**JHR**	**DOC**	**JHR**	**DOC**
PM_2.5_	**0.80** (0.64–0.97) ***	22.03%	0.80 (0.64–0.97)	22.02%	**0.79** (0.63–0.96) ***	22.03%	**0.79** (0.63–0.96) ***	22.02%
O_3_		51.52%		51.53%		51.52%		51.52%
NO_2_		18.38%		18.38%		18.38%		18.38%
SO_2_		8.08%		8.07%		8.07%		8.08%

Notes: JHR, calculated using the CRI, represent the relative hazards for a 10 µg/m^3^ increase in each pollutant compared with the scenario with no increase. Degree of contribution (DOC) indicates the percentage contribution of each pollutant to the overall JHRs. ***: p < 0.001; **: p < 0.01; and *: p < 0.05.

**Fig. 4:**
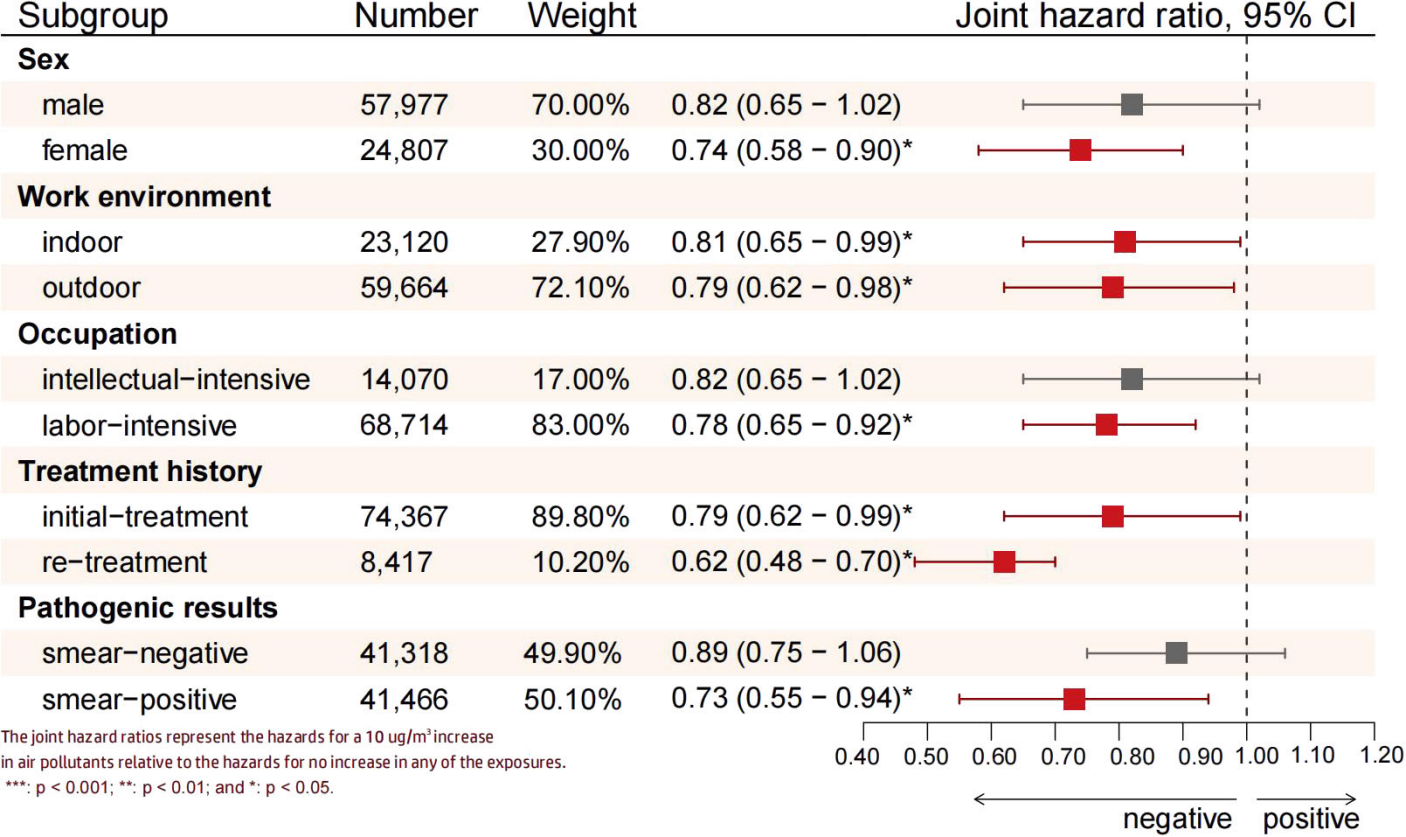
Combined risk of air pollutants across different subgroups.

Figure 4 illustrates the combined effect of air pollutants on PTB treatment outcomes across different subgroups. Outdoor workers exhibited higher risks associated with exposure to air pollutant mixtures (JHR = 0.79, 95% CI: 0.62–0.98) than indoor workers (JHR = 0.81, 95% CI: 0.65–0.99). Among patients with a history of PTB treatment, the adverse association was more pronounced in the retreatment group (JHR = 0.62, 95% CI: 0.48–0.70). Additionally, female patients (JHR = 0.74, 95% CI: 0.58–0.90), those engaged in labor-intensive work (JHR = 0.78, 95% CI: 0.65–0.92), and patients with positive etiological results (JHR = 0.73, 95% CI: 0.55–0.94) were more vulnerable to the negative effects of air pollutant mixtures.

### 3.4 Effect modification of greenspace

Table 3 presents the effect modification of greenspace on the combined risk associated with air pollutant mixtures. The analysis showed a reduced risk in areas with moderate levels of greenspace (T2, JHR = 0.81, 95% CI: 0.62–0.98) compared with the third quantile model (T3, JHR = 0.68, 95% CI: 0.52–0.83; Table 3). However, no significant results were observed for areas with low levels of greenspace (T1, JHR = 0.82, 95% CI: 0.66–1.01). Sensitivity analysis indicated slightly attenuated associations when using a 500 m buffer to calculate greenspace coverage (Table S9). The inclusion of migrant individuals in the analysis yielded consistent effect estimates (Table S10). Moreover, after excluding farmland from the definition of greenspace, the negative association between air pollutant mixtures and PTB treatment success became statistically significant in areas with low levels of greenspace (HR = 0.79, 95% CI: 0.72–0.87). Meanwhile, this association remained stable in areas with moderate and high levels of greenspace (HR = 0.81 and 0.76, respectively; Table S11).

**Table 3:** JHRs of four air pollutants (PM_2.5_, NO_2_, SO_2_, and O_3_) across different levels of greenspace.

**Variable**	**Tertile 1 (range 0.00%–32.43%)**		**Tertile 2 (range 32.43%–79.04%)**		**Tertile 3 (range 79.04%–100%)**	

	**JHR**	**DOC**	**JHR**	**DOC**	**JHR**	**DOC**
PM_2.5_	0.82 (0.66–1.01)	22.58%	0.81 (0.62–0.98) *	22.02%	0.68 (0.52–0.83) *	21.40%
O_3_		49.70%		51.52%		53.44%
NO_2_		20.15%		18.38%		16.51%
SO_2_		7.56%		8.08%		8.65%

Notes: JHR, calculated using the CRI, represents the relative hazards for a 10 µg/m^3^ increase in each pollutant compared with the scenario with no increase. DOC indicates the percentage contribution of each pollutant to the overall JHRs.

## 4. Discussion

### 4.1 Individual effect of air pollutants on PTB treatment

Our findings suggest that long-term exposure to PM_2.5_, NO_2_, and O_3_ is significantly negatively associated with the successful treatment outcomes of patients with PTB, which may be attributed to weakened immune defenses and decreased adherence to anti-TB therapy. Toxicological studies indicate that exposure to these pollutants above specific thresholds can impair the body’s immune response to *M.tb*, leading to oxidative stress and inflammatory reactions in the lungs. Such physiological changes may not only extend treatment duration but also elevate mortality risks [44–46]. Additionally, long-term exposure to PM_2.5_ and NO_2_ could elevate psychological stress during anti-TB treatment by triggering neuroinflammation [47, 48]. This stress may further increase the likelihood of developing multidrug-resistant TB, particularly when compounded by factors such as irregular medication use or malnutrition [49, 50].

Furthermore, our findings reveal a positive correlation between SO_2_ exposure and successful PTB treatment outcomes. This finding contrasts with heterogeneous epidemiological evidence observed across different regions, which may be attributable to variations in geographical exposure levels and population susceptibility [51]. Time-series studies in Hefei (mean SO_2_: 13.80 µg/m^3^) and Ningbo (25 µg/m^3^), China, reported inverse correlations between SO_2_ levels and TB clinic visits [52, 53], whereas a cohort study in Shandong province, China, found protective effects against multidrug-resistant TB at an SO_2_ concentration of 31 µg/m^3^ [53]. In contrast, a 12-year study in Shenyang province, China did not find a significant association between SO_2_ levels (mean SO_2_:63 µg/m^3^) and respiratory mortality [53].

In Zhejiang province from 2015 to 2019, the annual mean SO_2_ concentration (14.26 µg/m^3^) was significantly lower than in comparative regions (Ningbo: 25; Shenyang: 63; national average: 23.1 µg/m^3^), and consistently below the WHO guidelines (40 µg/m^3^). This observed association may be attributed to the bacteriostatic effects at subtoxic concentrations of SO_2_ (5.36–40.43 µg/m^3^) [54], as experimental evidence suggests that low-dose SO_2_ can disrupt *M.tb* by causing oxidative damage to lipids, proteins, and DNA [55]. Geographical source variations further contextualize these findings: in Zhejiang province, SO_2_ emissions primarily originate from marine fuel combustion with low sulfur content, in contrast to coal-dominated emissions in inland cities [56, 57]. While this mechanism is plausible, residual confounding from unmeasured factors such as disparities in healthcare access necessitates further confirmation through personal exposure monitoring cohorts.

### 4.2 Combined effect of multiple air pollutants on PTB treatment outcomes

To the best of our knowledge, this is one of the first large, population-based epidemiological studies examining the association between air pollutant mixtures and PTB treatment outcomes. While previous research has highlighted the role of treatment plans and patient compliance in PTB treatment success [58], the impact of modifiable environmental factors, such as ambient air pollution, has remained unclear. This study assesses the combined effects of multiple air pollutants and demonstrates their negative association with PTB treatment success, with JHR of 0.79 (95% CI: 0.63–0.96). The observed harmful effect can be attributed to the complex interactions among various air pollutants, including the physical adsorption of particulate matter and chemical reactions between pollutants that amplify inflammation and oxidative stress within the respiratory system. Consequently, these physiological responses may interfere with the absorption and metabolism of anti-TB medications, thereby reducing treatment efficacy [45]. Additionally, these pollutants may impact adherence to PTB treatment. Exposure to air pollution has been associated with increased psychological stress [47, 48], which can hinder the ability of patients to consistently follow their treatment regimens. These challenges in adherence may result from stress-induced impairments in cognitive function and behavioral changes that are crucial for the successful management of PTB therapy.

O_3_ and PM_2.5_ are identified as the primary contributors to the combined effect of air pollutants, with O_3_ accounting for 51.52% and PM_2.5_ for 22.02%, together comprising approximately 73.54% of the total impact. While previous studies have primarily focused on the associations between PM_2.5_, NO_2_, and TB outcomes [15, 16], our findings underscore the critical role of O_3_ in impeding TB treatment success in Zhejiang Province. This divergence aligns with the province’s evolving air quality profile: despite achieving a 9.5% annual reduction in PM_2.5_ concentrations from 2013 to 2017 [59], ground-level O_3_ pollution has intensified, with annual means increasing from 139 µg/m^3^ to 167 µg/m^3^ (+20.1%, [60]), consistently surpassing WHO peak-season thresholds of 60 µg/m^3^ [59]. The 24-month exposure window likely captured the cumulative effects of O_3_-induced respiratory immunosuppression. This is particularly relevant given that (i) O_3_’s well-documented ability to impair alveolar macrophage function through oxidative stress pathways, and (ii) secondary pollutants are becoming increasingly prominent under stringent PM_2.5_ control measures. O_3_ exacerbates bronchial hyperreactivity and compromises immune responses through interactions with the lungs, potentially leading to prolonged disease duration and reduced likelihood of successful treatment outcomes [61, 62]. Correspondingly, implementing synergistic control measures for PM_2.5_ and O_3_ could be a cost-effective strategy to improve PTB treatment success, particularly in areas where rising O_3_ levels are observed due to hydroperoxyl radicals resulting from extensive air pollution prevention and control measures targeting PM_2.5_ [63, 64].

Additionally, our findings suggest that patients with a history of PTB and those engaged in outdoor occupations are more susceptible to the detrimental effects of air pollutant mixtures during their treatment. This increased vulnerability among individuals with a history of PTB retreatment may be due to compromised immune systems and impaired lung function [6]. Moreover, outdoor workers are more likely to inhale a mixture of air pollutants [65], which can accumulate in the lungs over time and further impair lung function, particularly during PTB treatment.

### 4.3 Effect modification of greenspace

A significant modifying effect of greenspace on the association between multiple air pollutants and PTB treatment outcomes is observed, particularly in areas with moderate to high levels of greenspace. Several potential explanations may account for these findings. First, greenspace can effectively reduce the concentration of multiple air pollutants through the ecological functions of vegetation, which mitigates their negative effects on PTB treatment [22, 28, 66]. Second, greenspace has the potential to mitigate interactions among different air pollutants by lowering air temperatures [27, 28], which in turn reduces the production of secondary pollutants that could harm respiratory health. Third, increasing exposure to greenspace can provide patients with health benefits associated with nature, such as greater engagement in physical activities (e.g., walking and cycling), improved psychophysiological stress recovery, and enhanced social cohesion [67, 68].

Additionally, the impact of air pollutant mixtures on PTB treatment success is less pronounced in areas with moderate levels of greenspace than in those with high levels of greenspace, which contrasts with previous studies [69]. This discrepancy may be attributed to variations in greenspace characteristics and utilization patterns among patients with PTB across different regions. Urban areas tend to have moderate levels of greenspace, whereas rural and remote locations are more likely to feature forests with higher greenspace coverage [70]. However, despite the potential for extensive greenspace in rural and remote areas to mitigate the harmful effects of multiple air pollutants, these areas often lack essential amenities required to support patient activities during PTB treatment [71, 72]. These findings suggest a non-linear relationship regarding the modifying effect of greenspace on the association between air pollution and PTB treatment outcomes, which highlights the need for further investigation in future studies. Nonetheless, promoting exposure to greenspace, particularly in areas with moderate or high coverage, represents a cost-effective, non-medical intervention for improving PTB treatment outcomes.

### 4.4 Strengths and limitations

This study has several strengths. First, the use of a well-documented PTB treatment database, which comprises 82,784 cases, ensured robust and reliable findings that can provide valuable insights for other high-burden PTB regions. Second, the investigation into the combined effects of air pollutants on PTB treatment outcomes, along with the evaluation of the relative contribution of each pollutant, offers valuable guidance for policymakers in developing targeted and effective regulatory measures for ambient air pollutants. Furthermore, this study addresses a gap in existing research, which has largely been driven by single-pollutant frameworks, by exploring the modifying effects of greenspace in the context of combined pollutant exposure. These findings highlight the need for further investigation into the mechanisms underlying how greenness interacts with air pollutant mixtures, so as to inform more comprehensive environmental and public health policies.

This study also has some limitations. First, the analysis did not include the composition of particulate matter, which may affect PTB treatment outcomes and potentially limit the comprehensive assessment of exposure to air pollutant mixtures in real-world settings. Second, although excluding migrant populations was essential to minimize residential self-selection bias and emphasize long-term environmental exposures, this approach may constrain the generalizability of our findings to mobile populations. Future research could incorporate geolocated lifetime mobility trajectories or develop migrant-specific exposure algorithms that account for multi-city residency patterns, thereby enhancing exposure assessments for transient cohorts. Third, although the improvement in O_3_ and SO_2_ exposure data resolution from 10 km (2015–2018) to 1 km in 2019, methodological consistency in fusion techniques and monitoring networks ensures that this transition does not introduce bias. Future studies should adopt uniform 1-km resolution analyses to further enhance the precision of exposure assessments. Fourth, we recognize that our single-exposure-window design has limitations in distinguishing between acute versus chronic pollution effects. Future studies could utilize distributed lag models with monthly exposure lags to better elucidate critical exposure periods while accounting for pollutant collinearity. Fifth, although cropland, forest, grassland, and shrub areas are commonly used as indicators for greenspace exposure, they primarily reflect the extent of vegetation rather than other critical greenspace features such as plant structures and green infrastructure, which can significantly modify the association between air pollutant mixtures and PTB treatment outcomes. Further research is needed to investigate the interactions between different types of greenspace and air pollutants, as well as their subsequent impacts on health outcomes. Sixth, urban-rural stratification was not feasible due to limitations in historical boundary data. Future studies could enhance urbanicity classification by incorporating nighttime light data or population density metrics.

## 5. Conclusions

In this large-scale, population-based study, we found that long-term exposure to air pollutant mixtures impeded successful PTB treatment outcomes. O_3_ was consistently identified as the most influential air pollutant, followed by PM_2.5_ and NO_2_. Furthermore, exposure to moderate and high levels of greenspace during the PTB treatment period could mitigate the harmful effects of air pollutant mixtures. These findings highlight the importance of integrating strategies to reduce the combined effects of air pollutants and enhance greenspace into future PTB treatment policies.

## Supplementary information

**Additional file 1:** **Table S1.** Comparison of baseline characteristics between the migrant-included group and migrant-excluded group. **Table S2.** Definition of PTB treatment outcomes. **Table S3.** Baseline characteristics of air pollution and greenspace. **Table S4.** Results of collinearity test for variance inflation factor. **Table S5.** Associations between air pollution and PTB treatment using multi-exposure models based on stepwise regression (quintile analysis). **Table S6.** Associations of individual air pollutants with PTB treatment considering exposure within 500 m and 1,250 m buffers of air pollutants exposure. **Table S7.** Associations of individual air pollutants with PTB treatment excluding patients with drug-resistant PTB using multi-exposure models. **Table S8.** Associations between individual air pollutants and PTB treatment outcomes in multi-exposure models (Migrant-included model). **Table S9.** The joint hazard ratio (JHR) of four air pollutants based on the level of greenspace (tertiles) within a 500 m buffer zone of greenspace exposure. **Table S10.** JHRs and degree of contribution (DOC) of air pollutants (Migrant-included model). **Table S11.** The joint hazard ratio (JHR) of four air pollutants based on the level of greenspace (tertiles) within a 1,250 m buffer zone of greenspace exposure (excluding farmland). **Figure S1.** Spearman ranks correlations among air pollution, greenspace, and temperature.

## Declarations

### Availability of data and materials

All data generated or analysed are included in this article and its supplementary information files. The corresponding authors can provide data upon reasonable request after completing all studies and sub-studies.
