# Supplementary material for: Can greenspace modify the combined effects of multiple air pollutants on pulmonary tuberculosis treatment outcomes? An empirical study conducted in Zhejiang Province, China
Source: Environ Health Prev Med. 2025 May 2;30:31. doi: 10.1265/ehpm.24-00381 (PMC12062829; doi:10.1265/ehpm.24-00381)
Supplement: Supplementary file 1 — Additional file 1: Table S1. Comparison of baseline characteristics between the migrant-included group and migrant-excluded group. Table S2. Definition of PTB treatment outcomes. Table S3. Baseline characteristics of air pollution and greenspace. Table S4. Results of collinearity test for variance inflation factor. Table S5. Associations between air pollution and PTB treatment using multi-exposure models based on stepwise regression (quintile analysis). Table S6. Associations of individual air pollutants with PTB treatment considering exposure within 500 m and 1,250 m buffers of air pollutants exposure. Table S7. Associations of individual air pollutants with PTB treatment excluding patients with drug-resistant PTB using multi-exposure models. Table S8. Associations between individual air pollutants and PTB treatment outcomes in multi-exposure models (Migrant-included model). Table S9. The joint hazard ratio (JHR) of four air pollutants based on the level of greenspace (tertiles) within a 500 m buffer zone of greenspace exposure. Table S10. JHRs and degree of contribution (DOC) of air pollutants (Migrant-included model). Table S11. The joint hazard ratio (JHR) of four air pollutants based on the level of greenspace (tertiles) within a 1,250 m buffer zone of greenspace exposure (excluding farmland). Figure S1. Spearman ranks correlations among air pollution, greenspace, and temperature. [file ehpm-30-031-s001.docx]

# Supplementary Materials

**Table S1.** Comparison of baseline characteristics between the migrant-included group and migrant-excluded group.

**Table S2.** Definition of PTB treatment outcomes.

**Table S3.** Baseline characteristics of air pollution and greenspace.

**Table S4.** Results of collinearity test for variance inflation factor.

**Table S5.** Associations between air pollution and PTB treatment using multi-exposure models based on stepwise regression (quintile analysis).

**Table S6.** Associations of individual air pollutants with PTB treatment considering exposure within 500 m and 1,250 m buffers of air pollutants exposure.

**Table S7.** Associations of individual air pollutants with PTB treatment excluding patients with drug-resistant PTB using multi-exposure models.

**Table S8.** Associations between individual air pollutants and PTB treatment outcomes in multi-exposure models (Migrant-included model).

**Table S9.** The joint hazard ratio (JHR) of four air pollutants based on the level of greenspace (tertiles) within a 500 m buffer zone of greenspace exposure.

**Table S10.** JHRs and degree of contribution (DOC) of air pollutants (Migrant-included model).

**Table S11.** The joint hazard ratio (JHR) of four air pollutants based on the level of greenspace (tertiles) within a 1,250 m buffer zone of greenspace exposure (excluding farmland).

**Figure S1.** Spearman ranks correlations among air pollution, greenspace, and temperature.

| Table S1. Comparison of baseline characteristics between the migrant-included group and migrant-excluded group. | | | | |
| --- | --- | --- | --- | --- |
| **Patient characteristics** | **Migrant-included group (n = 131,498, %)** | **Migrant-excluded group (n = 82,784, %)** | **X^2^** | **P-Value** |
| **Sex** |  |  |  |  |
| Male | 90,677 (68.96%) | 24,807 (70.03%) | 28.20 | <0.001 |
| Female | 40,821 (31.04%) | 57,977 (29.97%) |  |  |
| **Age** |  |  |  |  |
| <=18 | 5,339 (4.06%) | 2,780 (3.36%) | 3,125.99 | <0.001 |
| >18，<=60 | 85,231 (64.82%) | 44,483 (53.73%) |  |  |
| >60 | 40,928 (31.12%) | 35,521 (42.91%) |  |  |
| **Occupation** |  |  |  |  |
| Labor-Intensive | 110,754 (84.22%) | 68,714 (83.00%) | 56.51 | <0.001 |
| Intellect-Intensive | 20,744 (15.78%) | 14,070 (17.00%) |  |  |
| **Work Environment** |  |  |  |  |
| Indoor | 37,889 (28.81%) | 23,120 (27.93%) | 19.87 | <0.001 |
| Outdoor | 93,609 (71.19%) | 59,664 (72.07%) |  |  |
| **Treatment Type** |  |  |  |  |
| Initial treatment | 119,924 (91.20%) | 74,367 (89.83%) | 113.95 | <0.001 |
| Retreatment | 11,574 (8.80%) | 8,417 (10.17%) |  |  |
| **Pathogenic results** |  |  |  |  |
| Smear-negative | 74,739 (56.84%) | 41,466 (50.09%) | 1,059.93 | <0.001 |
| Smear-positive | 56,759 (43.16%) | 41,318 (49.91%) |  |  |
| **Drug-susceptibility** |  |  |  |  |
| Drug resistance | 2,170 (2.62%) | 3,144 (2.39 %) | 16.18 | <0.001 |
| Non-drug resistance | 80,614 (97.38%) | 128,354 (97.61%) |  |  |

| Table S2. Definition of PTB treatment outcomes. | | |
| --- | --- | --- |
| **Treatment Outcomes** | | **Definition** |
| Successful Outcomes | Cured | Patients with smear-positive PTB who have completed the entire treatment course and have two consecutive negative smear results, with one occurring after the completion of therapy. |
|  | Treatment completed | Patients with smear-negative PTB who have finished the recommended treatment regimen and either have a negative sputum smear microscopy result or do not receive a smear examination after completion of therapy. Patients with smear-positive PTB who have completed the prescribed treatment and do not receive smear examination after completion of therapy, but the latest sputum smear result was negative. |
| Unsuccessful Outcomes | Treatment failed | PTB patients with positive sputum smear or culture results at the end of the 5th month or later during treatment. |
|  | Died | PTB patients who died from any cause before or during treatment. |
|  | Loss to follow up | PTB patients who fail to initiate or experience treatment interruption for two consecutive months or more. |

Table S3. Baseline characteristics of air pollution and greenspace**.**

| Table S3a. Baseline characteristics by greenspace (%), PM_2.5_ (µg/m^3^), NO_2_, SO_2_, and O_3_ assigned to all patients (n=82,784). | | | | | | |
| --- | --- | --- | --- | --- | --- | --- |
| **Full cohort** | **SO_2_** | **NO_2_** | **O_3_** | **PM_2.5_** | **PM_10_** | **Greenspace 1,250 m** |
| **Min.** | 5.36 | 9.39 | 65.38 | 19.47 | 31.35 | 0.09% |
| **1st Qu.** | 9.80 | 27.70 | 87.01 | 32.85 | 51.15 | 28.01% |
| **Median** | 13.00 | 34.15 | 91.33 | 38.47 | 58.70 | 58.04% |
| **Mean** | 14.26 | 34.08 | 91.98 | 39.63 | 59.76 | 55.43% |
| **3rd Qu.** | 17.66 | 39.93 | 95.75 | 45.13 | 67.50 | 83.71% |
| **Max.** | 40.43 | 61.29 | 122.03 | 70.72 | 103.10 | 100.00% |

Table S3b. Baseline characteristics by greenspace (%), PM_2.5_ (µg/m^3^), NO_2_, SO_2_, and O_3_ across the three tertiles of greenspace within a 1,250 m buffer.

|  | **SO_2_** | **NO_2_** | **O_3_** | **PM_2.5_** | **PM_10_** | **Greenspace 1,250 m** |
| --- | --- | --- | --- | --- | --- | --- |
| **Greenspace Tertile 1 (n=27,595)** |  |  |  |  |  |  |
| Min. | 5.36 | 12.57 | 65.38 | 19.47 | 31.35 | 0.09% |
| 1st Qu. | 9.89 | 33.76 | 89.06 | 35.63 | 56.74 | 8.71% |
| Median | 12.88 | 38.52 | 92.71 | 41.34 | 63.65 | 17.76% |
| Mean | 14.19 | 37.00 | 91.28 | 42.51 | 64.43 | 18.42% |
| 3rd Qu. | 17.35 | 44.43 | 97.70 | 48.47 | 71.58 | 28.01% |
| Max. | 40.43 | 61.29 | 122.03 | 70.71 | 103.27 | 38.14% |
| **Greenspace Tertile 2 (n=27,595)** |  |  |  |  |  |  |
| Min. | 5.35 | 12.50 | 65.91 | 19.47 | 33.05 | 38.14% |
| 1st Qu. | 9.64 | 28.74 | 88.08 | 33.34 | 52.01 | 48.22% |
| Median | 12.78 | 34.42 | 91.82 | 38.74 | 59.35 | 58.04% |
| Mean | 14.21 | 34.30 | 92.56 | 39.88 | 60.33 | 57.70% |
| 3rd Qu. | 17.62 | 39.68 | 96.22 | 45.26 | 67.70 | 67.26% |
| Max. | 40.31 | 59.53 | 121.92 | 68.33 | 98.90 | 75.89% |
| **Greenspace Tertile 3 (n=27,594)** |  |  |  |  |  |  |
| Min. | 5.35 | 9.39 | 68.52 | 20.32 | 31.35 | 75.89% |
| 1st Qu. | 9.87 | 23.65 | 85.90 | 30.39 | 46.95 | 83.72% |
| Median | 13.32 | 28.50 | 89.44 | 35.53 | 53.40 | 91.10% |
| Mean | 14.37 | 28.95 | 89.91 | 36.50 | 54.52 | 90.18% |
| 3rd Qu. | 18.00 | 34.25 | 93.01 | 41.20 | 60.95 | 97.12% |
| Max. | 38.80 | 56.40 | 121.02 | 66.15 | 92.75 | 100.00% |

| Table S4. Results of collinearity test for variance inflation factors. | | |
| --- | --- | --- |
| Variable | VIF | 1/VIF |
| PM_2.5_ | 5.74 | 0.1743 |
| SO_2_ | 3.54 | 0.2827 |
| NO_2_ | 3.39 | 0.2954 |
| Work Environment | 2.22 | 0.4503 |
| Occupation | 2.19 | 0.4571 |
| Greenspace | 1.65 | 0.6077 |
| O_3_ | 1.42 | 0.7066 |
| Pathogenic results | 1.09 | 0.9183 |
| Age | 1.08 | 0.9260 |
| Drug-susceptibility | 1.03 | 0.9748 |
| Treatment History | 1.02 | 0.9786 |
| Sex | 1.02 | 0.9812 |
| Mean VIF | 2.11 | -- |

| Table S5. Associations between air pollution and PTB treatment using multi-exposure models based on stepwise regression (quintile analysis). | | | | |
| --- | --- | --- | --- | --- |
| **Variable** | **HRs (95% CI)** | | | |
|  | **Model 1** | **Model 2** | **Model 3** | **Model 4** |
| **PM_2.5_** | **reference** |  |  |  |
| Q2 | 0.99 (0.97-1.01) | 0.99 (0.97-1.01) | 0.99 (0.97-1.01) | 0.99 (0.97-1.01) |
| Q3 | 1.01 (0.99-1.04) | 1.01 (0.98-1.03) | 1.02(0.99-1.04) | 1.01(0.99-1.03) |
| Q4 | 1.01(0.98-1.03) | 1.00 (0.98-1.03) | 1.01 (0.98-1.04) | 1.01 (0.98-1.04) |
| Q5 | **0.95 (0.92-0.98) **** | **0.94 (0.91-0.97) ***** | **0.96 (0.92-0.99) *** | **0.95 (0.92-0.99) **** |
| **O_3_** | **reference** |  |  |  |
| Q2 | **0.98 (0.96-0.99) *** | **0.98 (0.96-0.99) *** | 0.99 (0.97-1.01) | **0.97 (0.95-0.99) **** |
| Q3 | **0.89 (0.88-0.91) ***** | **0.89 (0.88-0.91) ***** | **0.90 (0.89-0.92) ***** | **0.89 (0.87-0.9) ***** |
| Q4 | **0.92 (0.9-0.94) ***** | **0.91 (0.9-0.93) ***** | **0.93 (0.91-0.95) ***** | **0.91 (0.89-0.93) ***** |
| Q5 | **0.89 (0.87-0.9) ***** | **0.88 (0.86-0.9) ***** | **0.89 (0.87-0.91) ***** | **0.89 (0.87-0.9) ***** |
| **SO_2_** | **reference** |  |  |  |
| Q2 | **1.12 (1.1-1.14) ***** | **1.12 (1.1-1.14) ***** | **1.11 (1.09-1.13) ***** | **1.13 (1.11-1.15) ***** |
| Q3 | **1.19 (1.17-1.22) ***** | **1.20 (1.17-1.22) ***** | **1.19 (1.16-1.22) ***** | **1.21 (1.18-1.23) ***** |
| Q4 | **1.28 (1.24-1.31) ***** | **1.28 (1.25-1.31) ***** | **1.29 (1.26-1.33) ***** | **1.30 (1.26-1.33) ***** |
| Q5 | **1.57 (1.53-1.62) ***** | **1.58 (1.54-1.63) ***** | **1.54 (1.5-1.59) ***** | **1.61 (1.56-1.65) ***** |
| **NO_2_** | **reference** |  |  |  |
| Q2 | **0.97 (0.95-0.98) ***** | **0.97 (0.95-0.98) ***** | **0.98 (0.96-0.99) *** | **0.96 (0.94-0.98) ***** |
| Q3 | 0.99 (0.97-1.01) | 0.99 (0.97-1.01) | 0.99 (0.97-1.01) | 0.98 (0.96-1) |
| Q4 | **0.96 (0.94-0.99) **** | **0.96 (0.94-0.98) ***** | **0.95 (0.93-0.98) ***** | **0.95 (0.93-0.98) ***** |
| Q5 | **0.91 (0.88-0.93) ***** | **0.91 (0.88-0.93) ***** | **0.90 (0.87-0.92) ***** | **0.90 (0.87-0.92) ***** |

Notes: Model 1 solely incorporated air pollutants without any adjustments. Model 2 adjusted for age and sex, while Model 3 additionally controlled for individual clinical variables, including treatment type, drug-susceptibility, and pathogen results. Model 4 further incorporated meteorological factors, socio-economic status, occupation type, and work environment. Model 4 represents the full model adjusting for all covariates. Q1-Q5: Quintiles 1 through 5 of air pollutant concentration. ***: p < 0.001; **: p <0.01; *: p <0.05.

| Table S6. Associations of individual air pollutants with PTB treatment considering exposure within 500 m and 1,250 m buffers of air pollutants exposure. | | | | | |
| --- | --- | --- | --- | --- | --- |
| **Variable** | **HRs (95% CI)** | **p-value** | **Variable** | **HRs (95% CI)** | **p-value** |
|  | **500 m-Model 1** |  |  | **1,250 m-Model 1** |  |
| **PM_2.5_** | 0.94 (0.93-0.96) | <0.001 | **PM_2.5_** | 0.96 (0.94-0.98) | <0.001 |
| **O_3_** | 0.98 (0.97-0.99) | 0.007 | **O_3_** | 0.98 (0.97-1.00) | 0.009 |
| **SO_2_** | 1.50 (1.47-1.54) | <0.001 | **SO_2_** | 1.48 (1.45-1.52) | <0.001 |
| **NO_2_** | 0.93 (0.92-0.95) | <0.001 | **NO_2_** | 0.93 (0.91-0.94) | <0.001 |
| **500 m-Model 2** | | | **1,250 m-Model 2** | | |
| **PM_2.5_** | 0.94 (0.93-0.96) | <0.001 | **PM_2.5_** | 0.96 (0.94-0.98) | <0.001 |
| **O_3_** | 0.98 (0.97-1.00) | 0.008 | **O_3_** | 0.98 (0.97-1.00) | 0.01 |
| **SO_2_** | 1.50 (1.47-1.54) | <0.001 | **SO_2_** | 1.49 (1.45-1.52) | <0.001 |
| **NO_2_** | 0.93 (0.92-0.95) | <0.001 | **NO_2_** | 0.93 (0.91-0.94) | <0.001 |
| **500 m-Model 3** | | | **1,250 m-Model 3** | | |
| **PM_2.5_** | 0.94 (0.92-0.96) | <0.001 | **PM_2.5_** | 0.95 (0.94-0.97) | <0.001 |
| **O_3_** | 0.98 (0.97-1.00) | 0.01 | **O_3_** | 0.98 (0.97-1.00) | 0.012 |
| **SO_2_** | 1.51 (1.47-1.55) | <0.001 | **SO_2_** | 1.49 (1.46-1.53) | <0.001 |
| **NO_2_** | 0.93 (0.92-0.95) | <0.001 | **NO_2_** | 0.92 (0.91-0.94) | <0.001 |
| **500 m-Model 4** | | | **1,250 m-Model 4** | | |
| **PM_2.5_** | 0.94 (0.92-0.96) | <0.001 | **PM_2.5_** | 0.95 (0.93-0.97) | <0.001 |
| **O_3_** | 0.98 (0.97-0.99) | 0.006 | **O_3_** | 0.98 (0.97-0.99) | 0.007 |
| **SO_2_** | 1.54 (1.5-1.58) | <0.001 | **SO_2_** | 1.52 (1.48-1.56) | <0.001 |
| **NO_2_** | 0.92 (0.91-0.94) | <0.001 | **NO_2_** | 0.91 (0.9-0.93) | <0.001 |

Notes: HRs, hazard ratios with 95% confidence intervals (CIs) are calculated for each 10 µg/m³ increase of air pollutant exposure. Model 1 includes only air pollutants without adjustments. Model 2 is adjusted for age and sex, while Model 3 is additionally controlled for patient characteristics and treatment modalities variables, including treatment history, drug-susceptibility, and pathogenic results. Model 4 is additionally controlled for meteorological factors and socioeconomic status. Model 4 represents the full model and adjusts for all covariates.

| Table S7. Associations of individual air pollutants with PTB treatment excluding patients with drug-resistant PTB using multi-exposure models. | | | | | |
| --- | --- | --- | --- | --- | --- |
| **Model 1** | | | **Model 2** | | |
| **Variable** | **HRs (95% CI)** | **p-value** | **Variable** | **HRs (95% CI)** | **p-value** |
| **PM_2.5_** | 0.95 (0.93-0.97) | <0.001 | **PM_2.5_** | 0.95 (0.93-0.97) | <0.001 |
| **O_3_** | 0.98 (0.97-0.99) | 0.006 | **O_3_** | 0.98 (0.97-0.99) | 0.007 |
| **SO_2_** | 0.93 (0.92-0.95) | <0.001 | **SO_2_** | 0.93 (0.92-0.95) | <0.001 |
| **NO_2_** | 1.49 (1.46-1.53) | <0.001 | **NO_2_** | 1.49 (1.46-1.53) | <0.001 |
| **Model 3** | | | **Model 4** | | |
| **Variable** | **HRs (95% CI)** | **p-value** | **Variable** | **HRs (95% CI)** | **p-value** |
| **PM_2.5_** | 0.94 (0.93-0.96) | <0.001 | **PM_2.5_** | 0.94 (0.92-0.96) | <0.001 |
| **O_3_** | 0.98 (0.97-0.99) | 0.006 | **O_3_** | 0.98 (0.97-0.99) | 0.003 |
| **SO_2_** | 0.93 (0.92-0.95) | <0.001 | **SO_2_** | 0.92 (0.91-0.94) | <0.001 |
| **NO_2_** | 1.50 (1.47-1.54) | <0.001 | **NO_2_** | 1.53 (1.49-1.57) | <0.001 |

Notes: HRs, Hazard ratios with 95% confidence intervals (CIs) are calculated for each 10 µg/m³ increase in air pollutant exposure. Model 1 includes only air pollutants without adjustments. Model 2 is adjusted for age and sex, while Model 3 is additionally controlled for patient characteristics and treatment modalities variables, including treatment history, drug-susceptibility, and pathogenic results. Model 4 is additionally controlled for meteorological factors and socioeconomic status. Model 4 represents the full model and adjusts for all covariates.

Table S8. Associations between individual air pollutants and PTB treatment outcomes in multi-exposure models (Migrant-included model).

| **Model 1** | | | **Model 2** | | |
| --- | --- | --- | --- | --- | --- |
| Variable | HRs (95% CI) | p-value | Variable | HRs (95% CI) | p-value |
| PM_2.5_ | 0.95 (0.93-0.97) | <0.001 | PM_2.5_ | 0.94 (0.93-0.96) | <0.001 |
| O_3_ | 0.98 (0.97-0.99) | 0.004 | O_3_ | 0.98 (0.97-0.99) | 0.003 |
| SO_2_ | 1.49 (1.46-1.53) | <0.001 | SO_2_ | 1.50 (1.47-1.54) | <0.001 |
| NO_2_ | 0.93 (0.92-0.95) | <0.001 | NO_2_ | 0.94 (0.92-0.95) | <0.001 |
| **Model 3** | | | **Model 4** | | |
| Variable | HRs (95% CI) | p-value | Variable | HRs (95% CI) | p-value |
| PM_2.5_ | 0.94 (0.92-0.96) | <0.001 | PM_2.5_ | 0.94 (0.92-0.96) | <0.001 |
| O_3_ | 0.98 (0.97-0.99) | 0.007 | O_3_ | 0.98 (0.97-0.99) | 0.004 |
| SO_2_ | 1.52 (1.48-1.55) | <0.001 | SO_2_ | 1.53 (1.5-1.57) | <0.001 |
| NO_2_ | 0.93 (0.92-0.95) | <0.001 | NO_2_ | 0.92 (0.91-0.94) | <0.001 |

Notes: Model 1 included only air pollutants without any adjustments. Model 2 adjusted for demographic factors such as age and sex. Model 3 further controlled for individual clinical variables, including treatment type, drug susceptibility, and pathogen results. Model 4 incorporated additional factors such as meteorological factors, socioeconomic status, occupation type, and work environment, representing the fully adjusted comprehensive model.

| Table S9. The joint hazard ratio (JHR) of four air pollutants based on the level of greenspace (tertiles) within a 500 m buffer zone of greenspace exposure. | | | | | | |
| --- | --- | --- | --- | --- | --- | --- |
|  | **Greenspace 500 m (Tertile 1)** | | **Greenspace 500 m (Tertile 2)** | | **Greenspace 500 m (Tertile 3)** | |
| **Variable** | **JHR** | **DOC** | **JHR** | **DOC** | **JHR** | **DOC** |
| PM_2.5_ | **0.81 (0.66-0.99)** | 22.50% | 0.86 (0.70-1.09） | 22.02% | **0.74 (0.58-0.91）** | 21.51% |
| O_3_ |  | 49.98% |  | 51.56% |  | 53.18% |
| NO_2_ |  | 19.93% |  | 18.34% |  | 16.71% |
| SO_2_ |  | 7.58% |  | 8.08% |  | 8.61% |
| Note: JHR, calculated using the CRI, represent the relative hazards for a 10 µg/m3 increase in each pollutant compared with the scenario with no increase. Degree of contribution (DOC) indicates the percentage contribution of each pollutant to the overall JHRs. | | | | | | |

Table S10. JHRs and degree of contribution (DOC) of air pollutants (Migrant-included model).

| Variable | Model 1 | | Model 2 | | Model 3 | | Model 4 | |
| --- | --- | --- | --- | --- | --- | --- | --- | --- |
|  | JHR | DOC | JHR | DOC | JHR | DOC | JHR | DOC |
| PM_2.5_ | **0.86**  **(0.84-0.88)^**^** | 17.65% | **0.85**  **(0.82-0.86)^***^** | 18.75% | **0.85**  **(0.83-0.87)^***^** | 19.84% | **0.86**  **(0.83-0.89)^***^** | 18.49% |
| O_3_ |  | 48.18% |  | 47.73% |  | 47.87% |  | 48.37% |
| SO_2_ |  | 15.59% |  | 15.63% |  | 13.93% |  | 12.52% |
| NO_2_ |  | 18.58% |  | 17.89% |  | 18.37% |  | 20.62% |

Notes: Model 1 included only air pollutants without any adjustments. Model 2 adjusted for demographic factors such as age and sex. Model 3 further controlled for individual clinical variables, including treatment type, drug susceptibility, and pathogen results. Model 4 incorporated additional factors such as meteorological factors, socioeconomic status, occupation type, and work environment, representing the fully adjusted comprehensive model. ***: p < 0.001; **: p <0.01; *: p <0.05.

| Table S11. The joint hazard ratio (JHR) of four air pollutants based on the level of greenspace (tertiles) within a 1,250 m buffer zone of greenspace exposure (excluding farmland). | | | | | | |
| --- | --- | --- | --- | --- | --- | --- |
| **Variable** | **Greenspace 1250 m**  **(Tertile 1)** | | **Greenspace 1250 m**  **(Tertile 2)** | | **Greenspace 1250 m**  **(Tertile 3)** | |
|  | **JHR** | **DOC** | **JHR** | **DOC** | **JHR** | **DOC** |
| PM_2.5_ | **0.79**  **(0.72-0.87)***** | 25.06% | **0.81**  **(0.80-0.83)**  ******* | 15.36% | **0.76**  **(0.67-0.89)**  ******* | 19.18% |
| O_3_ |  | 45.45% |  | 43.71% |  | 52.72% |
| SO_2_ |  | 12.90% |  | 14.82% |  | 10.19% |
| NO_2_ |  | 16.59% |  | 26.11% |  | 17.90% |

Notes: ***: p < 0.001; **: p <0.01; *: p <0.05.


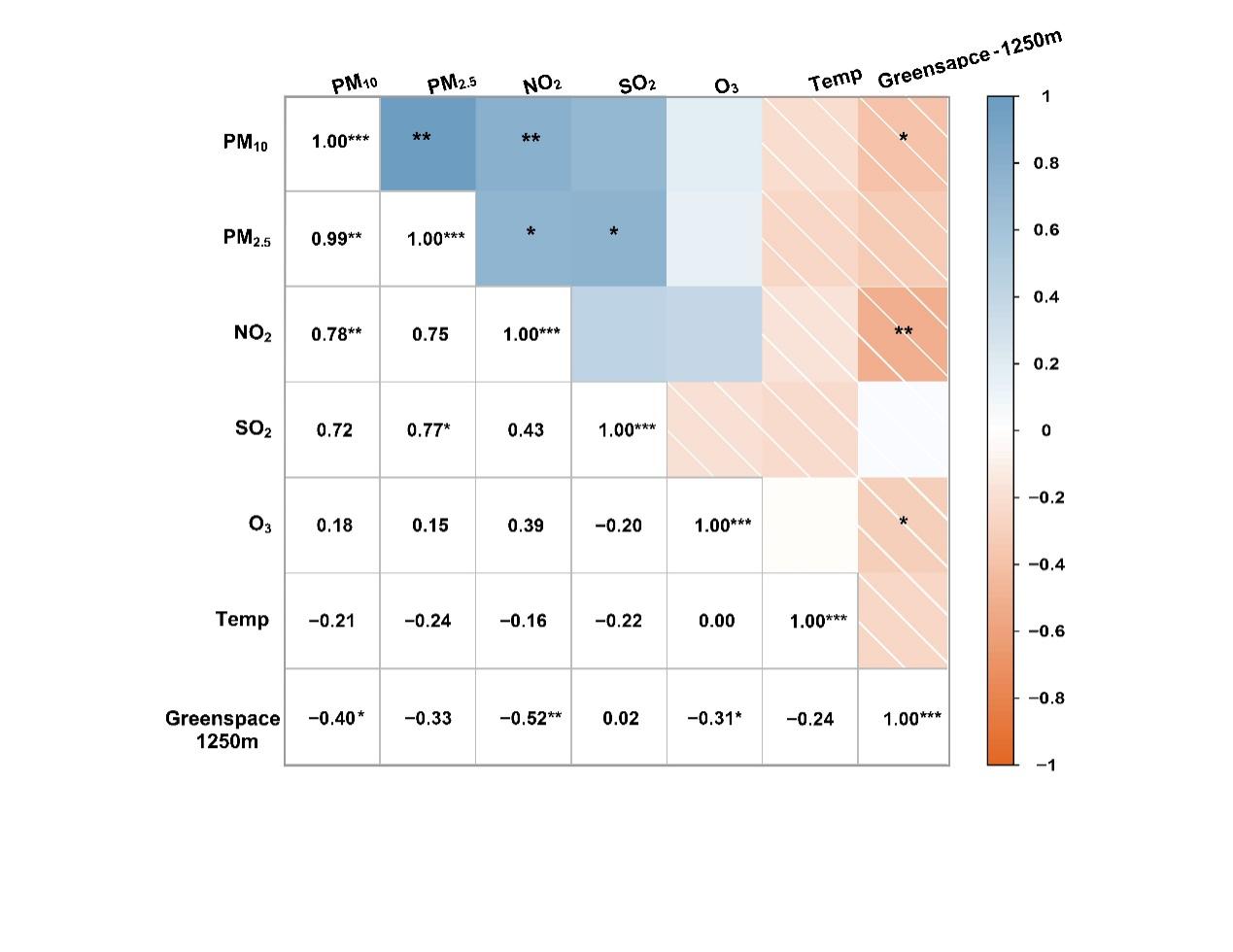


Figure S1. Spearman ranks correlations among air pollution, greenspace, and temperature.

Notes: Temp, temperature; Grenspace-1,250 m, surrounding greenspace in 1,250 m buﬀer zone. ***: p < 0.001; **: p < 0.01; *: p < 0.05.
